# Drivers and consequences of microbial community coalescence

**DOI:** 10.1093/ismejo/wrae179

**Published:** 2024-09-17

**Authors:** Xipeng Liu, Joana Falcão Salles

**Affiliations:** Microbial Ecology cluster, Genomics Research in Ecology and Evolution in Nature (GREEN), Groningen Institute for Evolutionary Life Sciences (GELIFES), University of Groningen, Nijenborgh 7, 9747 AG Groningen, The Netherlands; Ecologie Microbienne Lyon, Centre National de la Recherche Scientifique (CNRS) UMR5557, Bâtiment Grégoire Mendel, 69100 Villeurbanne, France; Microbial Ecology cluster, Genomics Research in Ecology and Evolution in Nature (GREEN), Groningen Institute for Evolutionary Life Sciences (GELIFES), University of Groningen, Nijenborgh 7, 9747 AG Groningen, The Netherlands

**Keywords:** dispersal, disturbance, invasion, community stability, interspecies interaction, microbial community dynamic, microbial inoculation

## Abstract

Microbial communities are undergoing unprecedented dispersion and amalgamation across diverse ecosystems, thereby exerting profound and pervasive influences on microbial assemblages and ecosystem dynamics. This review delves into the phenomenon of community coalescence, offering an ecological overview that outlines its four-step process and elucidates the intrinsic interconnections in the context of community assembly. We examine pivotal mechanisms driving community coalescence, with a particular emphasis on elucidating the fates of both source and resident microbial communities and the consequential impacts on the ecosystem. Finally, we proffer recommendations to guide researchers in this rapidly evolving domain, facilitating deeper insights into the ecological ramifications of microbial community coalescence.

## Introduction

**Figure 1:**
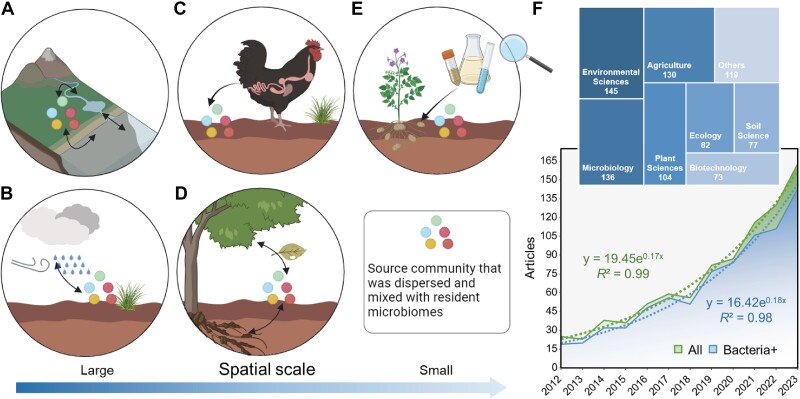
Examples of community coalescence in ecosystems (A–E) and the growing scientific interest in microbial artificial consortia in soils (F). Examples of microbiomes coalescence in different contexts: (**A**) aquatic systems [10,14–16], (**B**) atmospheric deposition [17–19], (**C**) animal guts [20–23], (**D**) phyllosphere and root systems [19,24], and (**E**) deliberate inoculations [25–27]. (**F**) Research on microbial consortium inoculation in soil systems has exponentially increased recently. “All” represents the total number of articles involving microbial consortia in soil, whereas “bacteria+” indicates those that specifically used bacteria as the consortium. The TreeMap shows the distribution pattern of the scientific research areas for these studies according to records from the web of science. We searched the web of science Core collection database using the search terms topic: (soil AND (microbial OR bacterial) AND (consortia OR consortium OR communit*) AND (inocula*)). All research articles published between 2012 and 2023 were included, resulting in 3011 papers. We then screened titles, abstracts, and full texts for performing the selection, excluding studies out of our scope, which returned a total of 866 studies. The research area for each study was recorded based on the information provided in the web of science database with the primary research field listed first in cases where multiple research fields were associated with a study.

Microorganisms can spread across various geographic distances, colonizing new environments and influencing native microbial communities. This phenomenon is described as microbial invasion and can be initiated by a single strain or a whole community, the latter also known as community coalescence or community-driven invasion [1–3]. Unlike the emigration of single species, which is mostly driven by anthropogenic activities (i.e. application of biological products used for plant growth promotion, biocontrol, and bioremediation), community coalescence represents a realistic and ever-present feature in natural ecosystems [3], frequently facilitated by water and wind flow, animal migration, or human activities ([4–6], Fig. 1). Community coalescence plays an important role in shaping microbial communities and ecosystem functions. For instance, evidence based on the distance-decay relationships [7–9] suggests that community coalescence in the soil can significantly regulate the resident community composition, at least on a short spatial scale. In aquatic environments, microbial community composition can change due to coalescences, as demonstrated by studies on the *in vitro* coalescences of lake communities and the observation of river ecosystems [10–13].

The mechanisms and ecological impacts of community coalescence have received limited attention. Community coalescence process involves the intricate blending of living and non-living elements [1, 2]. Biotic components, which refer to the potential interactions found within coalescent communities, are an important part of this mix. In the past, the effects of biotic components, such as microorganisms, were typically overlooked in cases such as the application of organic fertilizers or plant residues to soil or the mixing of rivers and seawater. Instead, abiotic factors were believed to govern the outcomes. However, growing evidence suggests that introduced microbes can play a significant role in regulating these processes and ultimately determining the outcome of the community succession [11,28–30]. A recent report showed that different source communities can have varying impacts on the same resident community by altering species diversity, community composition, and functionality and that these impacts become more pronounced over time due to alterations in soil moisture [31]. In other specific scenarios, such as when inoculating beneficial microbes into the soil or tracking pathogens’ migration and survival dynamics in water and soil, biotic factors in the coalescence process may be of primary concern. This is emphasized by the fact that community coalescence in the soil has reached an unprecedented level due to human-mediated intentional inoculations of microbial consortia ([32–34], Fig. 1).

Previous understanding of community coalescence is limited to the conceptual elaboration [1,3,35,36] and the speculation based on the empirical evidence from single-species invasions, which has been steered to focus on either the invader perspective (i.e. invader’s survival [37–41]) or the resident community perspective (i.e. impacts on community composition and function; [42–46]). However, the potential multitude of interactions in coalescent communities will likely generate complex effects on resident communities, given that species interactions regulate invasion mechanisms in single-species invaders [47]. Moreover, the recent development in ecological theory has clarified that community coalescence is integral to the assemblage of communities, including four fundamental processes: dispersal, selection, diversification, and drift [2,48,49]. The arrival of new species through community coalescence can subsequently lead to significant changes in resident communities over time, promoting secondary succession through autogenic processes [50,51], hence reshaping the balance between community assembly processes. The investigation of community coalescence could thus provide a unique perspective, linking four processes within the unified framework of microbial community assembly. This perspective could facilitate the prediction of dispersal/invasion outcomes in local and regional communities.

Considering the practical significance of community coalescence and its relevance in ecological theory, we propose a thorough and systematic overview, along with a mechanistic synthesis that integrates the mechanisms affecting both source communities (invaders) and native communities (residents or resident communities) [46]. Here, we summarize the coalescence process in the context of community assembly by uniting recent empirical evidence in both soil and aquatic systems. We synthesize the underlying mechanisms and embed them into an interaction-oriented mechanistic model, thereby providing a unified model for elucidating biotic and abiotic mechanisms. Based on these, we propose scientific questions and potential directions that implicate community coalescences’ practical and theoretical significance for guiding future research.

## Ecological stages of community coalescence

**Figure 2:**
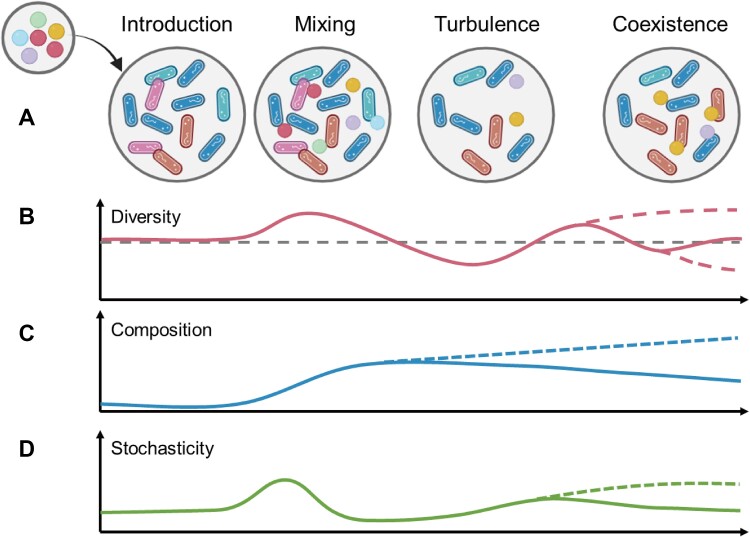
Process and patterns of bacterial community coalescence. (**A**) Four-step process of bacterial community coalescence. The coalescence starts with introducing a source (circular bacteria) community into a resident one (rod-shaped bacteria). During this phase, the introduced invaders interact with both residents and the environment. These interactions lead to complex dynamics and turbulence within the resident community, where mutual suppression between residents and invaders is likely to occur. The mixing and turbulence can occur quickly, potentially within hours or days [11,31,53]. The final step, termed “coexistence,” takes a relatively long time, likely tens of days, and involves the re-establishment of new interactions and rules within the coalesced community between survived invaders and residents and between survived resident taxa. (**B**–**D**) the shift patterns of diversity, composition, and assembly process (illustrated as the stochasticity governing the community assemblage) of the mixed community over time are shown. Dash lines indicate other potential patterns. Remarkable fluctuations in alpha and beta diversities may be detected in the mixing and turbulence stages [31,54]. The outcomes for bacterial diversity and composition during the coexistence period can vary, depending on the invaders’ survival and the suppression of residents [31,53,54]. The changes in stochasticity of coalesced communities may differ, influenced by the mixing of abiotic factors and the original status of both source and resident communities.

The ecological blueprint of the invasion process based on single-species invasion proposed in 2015 outlined four stages: introduction, establishment, growth and spread, and impact [47]. This represents the first time that comprehensively organized the entire process of microbial invasion from a dual perspective (including the invader and resident community) and describes the multiple barriers to the success of an invader. However, subsequent studies have demonstrated that even transient invaders, which cannot establish themselves, can leave (lasting) footprints in the recipient community [42,52]. This suggests that the invasion impacts can appear immediately after the introduction of the invader, regardless of its ability to grow and spread. Therefore, it is reasonable that (i) the impact of a community coalescence could be remarkable, even though some invasive members fail to establish successfully, and (ii) the consequences on invaders and residents should be distinguished. Indeed, community coalescence experiments in soil microcosms revealed that most invaders (99%) failed to survive [31]. Despite this, there were reciprocal influences, mainly in suppression, between invaders and native bacteria, highlighting the importance of microbial interactions before and after the coalescence [31]. These are not restricted to soil communities, as the coalescence of aquatic microbiomes also has similar consequences [11]. Based on these observations, we propose a four-step community coalescence process: introduction, mixing, turbulence, and coexistence (Fig. 2). This revised process aims to incorporate the scenario of transient invaders and emphasize the importance of microbial interactions pre/post-invasion.

Community coalescence begins with the introduction of invasive microbes into a new environment. This process, known as dispersal in the framework of microbial assembly, typically occurs passively [9,49]. During transportation, the invaders’ population density and ability to withstand various abiotic stresses are crucial in determining their chances of successful establishment and subsequent impacts [4,55].

Once the invaders arrive, they mix with the resident community. The mixing patterns can vary in impact on the invasion process and its consequences. For instance, bacteria carried on solid materials (such as soil aggregates, manure particles, and leaves) have a lower chance of mixing with resident soil microbiomes unless facilitated by other events like earthworm activities or tillage [3]. Conversely, bacteria in water or liquid inoculum (coalescence in aquatic systems) are more likely to be distributed homogeneously. However, the probability of interactions between invaders and native species can change depending on mixing patterns. For example, microbial inoculants immobilized by carriers in both terrestrial and aquatic systems (e.g., biochar and plant residue in soil systems [56–58]; suspended particles in aquatic systems [10]) can often survive longer and function better due to reduced competition. This can have significant consequences in the following phase, as longer survival time can lead to more substantial changes in the resident communities. Additionally, the carriers themselves can act as a substrate for the resident community, further impacting the ecosystem.

The turbulence stage of the coalescent community begins with the influence of invaders on resident species and the environment. An important feature of this stage might be dramatic fluctuations in species diversity of the coalesced community [31,59]. For instance, on average, 69% of constant resident species in soil were suppressed below the detection limit after invasion by microbial communities, resulting in a reduction of up to 65% in richness after 5 days [31]. Subsequently, the resident species that were not suppressed benefited from this and thrived in the coalesced community after invasions [31]. These results suggest that community coalescences can have distinct cascading effects on communities rather than isolated impacts on certain bacteria. This phenomenon also happens when soil microbiomes are affected by abiotic and biotic disturbances (e.g., heat shock, pH changes, and antibiotics) [60,61], especially when keystone taxa are disrupted [62]. Coalescence experiments in liquid media have shown that removing abundant taxa after mixing can lead to the emergence of otherwise extremely rare microbes [11]. Overall, scientific data indicate that bacterial community coalescence can trigger turbulence in coalesced communities, affecting diversity, composition, function, and interactions, whereas the coalescence pattern depends on the traits of two merging communities.

In the coexistence stage, two possible scenarios can occur: (i) new rules and interactions are established in the coalescent community (i.e. low resilience of the resident community leading to secondary succession), or (ii) the original interactions are restored when the invasion pressure slows down (i.e. high resilience of the resident community). The coexistence stage is characterized by new interactions appearing between invaders and residents, but also between resident species. On the one hand, facilitative interactions, such as cross-feeding or synergic interactions, can lead to a higher probability of invaders’ survival or a better functionality of resident species. In the case of invasion by a single species, e.g. the inoculant *Bacillus velezensis* SQR9 synergically interacts with the plant-beneficial indigenous *Pseudomonas stutzeri* in the cucumber rhizosphere through biofilm matrix components of *Bacillus*, promoting the plant growth and salt tolerance [63]. In contrast, in many cases, invasions steer the niche structure (i.e. metabolic profile) of communities invaded by single species or communities away from the original status [31,42]. This indicates that resident taxa are likely to reshape their metabolic interactions post-coalescence, with an increased preference for particular resources. It has been stated that the soil microbiome often lacks resilience to single-species invasions [64], even though the invaders failed to survive [42], so the establishment of new rules is highly anticipated in soil environments. In aquatic systems, even though the environment changes more rapidly (driven by environmental mixing), the survival of introduced species has been observed in large-scale watersheds [10] and in laboratory coalescence experiments [12,53]. However, the stability of abiotic environmental conditions in aquatic systems, influenced by seasonal forces (temperature and influx/outflow volume), largely determines the microbial dynamic and coexistence after coalescence [65,66].

## Community coalescence in the context of community assembly

The community coalescence that started with the dispersal of exotic pools [55] should be embedded into the theoretical framework of community assembly. Within this framework, the community can be governed by deterministic factors, including selection imposed by the abiotic environment (environmental filtering) and both antagonistic and synergistic species interactions, and stochastic factors, such as probabilistic dispersal and drift (e.g. unpredictable disturbance and random birth-death events) [67]. The deterministic process drives the community succession following an identical trajectory, whereas the stochastic process leads to unpredictable results of the community succession. Some accounts suggest that dispersal is mainly stochastic, especially in models that quantitatively assess the microbial community assembly process [68–71], thereby viewing the consequence of community coalescence as a “neutralizing force” in natural communities.

Empirical evidence suggests that community coalescence involves both deterministic and stochastic factors (Fig. 2D). For instance, the dispersal and introduction of invaders are relevant to both abiotic selection (environmental conditions during or after dispersal) and stochastic factors (random translocation of invaders through natural dispersal processes). Once the mixing occurs, the invaders start to interact with the resident species and often place selection pressure on resident communities [42,44,72]. Likewise, the empirical evidence from both soil and aquatic systems suggests that the community-level consequence of community coalescence (e.g. changes in alpha and beta diversity) is not random but rather predictable to some extent [31,53,60,73,74]. For instance, the co-selection between invaders and residents can affect the coalescence outcomes of *in vitro* mixed communities that widely exist among different communities [60] and cannot be altered by simply increasing the size of the source community [73]. Additionally, competition between invaders and resident species can suppress these competing resident community members, promoting the availability of the niches once occupied by them. These niches can then be occupied by species whose abundances were kept in check due to their lower competitive ability. A potential outcome of this scenario is an increase in the abundance of previously rare species or even an increase in alpha diversity as rarer species are detected [11,31,42]. This process may enhance the importance of drift, which is accompanied by microbial growth and mortality during niche occupation. Simultaneously, coalescence will change the existing niche segregation (reducing microbial interspecies competition to facilitate coexistence) among different species [31]. This may mechanistically alter the evolutionary dynamics of resident taxa [75] due to the absence of competitors. However, there is a great lack of empirical research about how coalescence affects drift and diversification and how these two processes influence the coalescence consequences.

Overall, community coalescence offers a unique window into the interplay of all four community assembly processes in local communities and metacommunities at a larger spatiotemporal scale. The selection pressure posed by the invaders is expected to affect residents’ drift and diversification directly. Furthermore, the coalescence may lead to an increased species diversity in coalescent communities due to the successful dispersal and establishment of invaders, as well as a decreased species diversity in coalescent communities due to the suppression and turbulence of residents. This may determine how microorganisms disperse from the coalescent community and the new outcomes of receiving microorganisms from other communities.

## Mechanistic model of community coalescences

Unraveling the mechanisms driving community coalescence is vital to understanding and predicting the consequences of this important and complex process. Next, we discuss key mechanisms in community coalescence while focusing on the fate of both invaders and resident communities and the potential impacts at the ecosystem level.

**Figure 3:**
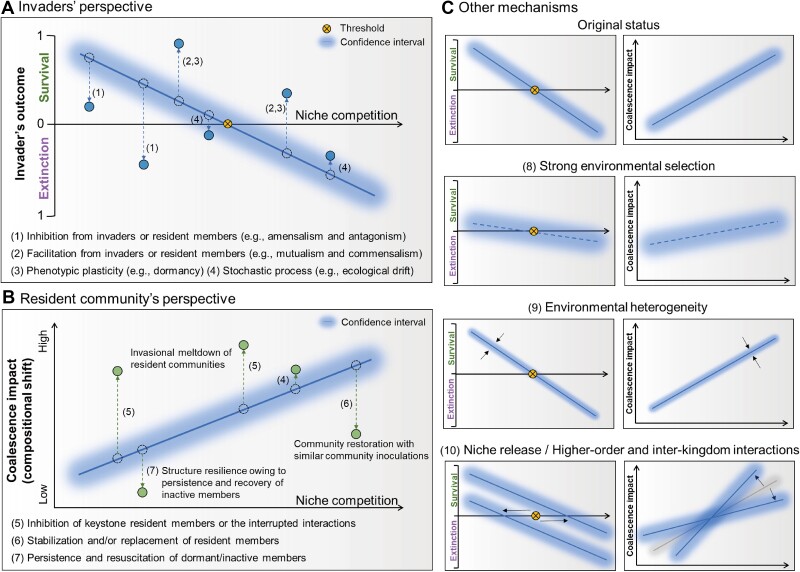
A conceptual overview of mechanisms regulating community coalescences. This model summarizes the mechanisms of bacterial community coalescences from (**A**) the invaders’ perspective and (**B**) the resident community’s perspective. Niche competition is considered the primary mechanism governing the survival of each invader [31], which might be interpreted by the phylogenetic distance between each invader and the resident community [78,79]. In both panels, the X axis represents niche competition, describing the theoretical competition between an invader and the resident community. The Y axis in the first panel indicates the theoretical probability of each invader surviving or becoming extinct after coalescence, whereas in the second panel, it represents the coalescence impact, such as compositional and functional shifts. The dashed and non-dashed circles indicate the theoretical and realistic statuses of outcomes, with numbers adjacent to arrows indicating different mechanisms governing coalescence outcomes. (**C**) Abiotic factors, higher-order interactions, and inter-kingdom interactions further modulate the mechanistic model.

For a natural community containing thousands of species, it is unimaginable to fully grasp the dynamics of coalescence progress and the mechanisms that govern the fate of each species. To understand the potential mechanisms that prevailed in bacterial communities when interacting with each other during coalescence, we introduce a mechanistic model that places biotic interactions at the forefront with additional consideration given to other mechanisms, such as environmental factors (Fig. 3). In this model, the primary mechanism considered is the competition for niches between invaders and resident species, considering the resident communities are at full carrying capacity, as this has been observed in both single-strain and community-driven invasions [12,31,38]. A negative correlation can be established between niche competition and the theoretical probability of each invader’s status, represented by the blue line, with the confidence interval representing the observational probability (Fig. 3). The threshold represents a certain level of niche competition that constrains invaders’ survival/extinction probability to zero. In addition to direct competition, interspecies interactions also have a significant impact on the empirical survival probability of an invader (blue points) compared to its theoretical status (dotted points) in the context of community coalescence. Among these interactions, amensalism, antagonism, and predator–prey interactions (No. 1, Fig. 3A) imposed on invaders may inhibit their survival despite lower niche competition [31,76], whereas mutualism and commensalism (No. 2, Fig. 3A) can be facilitative [63,77]. For example, although monitoring invaders’ survival in the soil is complicated, a recent meta-analysis suggests a synergistic effect between frequently used inoculums (e.g. *Bacillus* and *Pseudomonas*) contributed to the effectiveness of the consortium inoculation [77], which could indicate the role of facilitative interactions among invaders in living soil.

In addition to species interactions, other aspects related to the microbial life history traits, such as the species phenotypic plasticity, e.g. through dormancy (No. 3, Fig. 3A), might increase their chances of survival when facing biotic and abiotic selection during coalescence [80]. As discussed earlier, the outcomes of coalescences can also be driven by stochastic factors associated with drift processes, resulting in higher variation in the empirical response and a larger confidence interval (No. 4 in Fig. 3A). Drift would be more important when selection is weak, and the size of the invaders’/residents’ population is small [81]. The role of drift in shaping soil microbiomes in natural ecosystems and its impact on source communities, such as the survival of invaders, deserves further research.

From the resident community’s perspective, the resident species will also suffer from the biotic selection pressures exerted by invaders, known as a lose-lose phenomenon where both invaders and resident species are suppressed [31]. Therefore, the niche competition between the source and resident communities may govern the coalescence outcomes of compositional and functional shifts. The confidence interval represents the observational results, which can be influenced by stochastic factors (No. 4, Fig. 3B). There are several possible cases where coalescence impacts were changed outside the confidence interval. For example, the inhibition of keystone residents (the taxa that have a disproportionately large effect on the community and its natural environment [82]) and the disrupted interactions lead to invasional meltdown (No. 5, Fig. 3B) [62], whereas inoculations with taxonomic-similar (or functionally redundant) components may stabilize or restore the resident community against disturbances (No. 6, Fig. 3B) [54,59,83]. In addition, the coalescent community could be compositionally and functionally resilient due to the persistence and resuscitation of inactive resident species (No. 7, Fig. 3B) as it was suggested that dormant microorganisms act as a “seed bank” for preserving diversity and functions of microbial communities [84,85].

Abiotic environmental conditions can regulate the relationship between niche competition and invaders’ survival (invaders’ perspective, left panels in Fig. 3C) as well as the coalescence impacts (resident community’s perspective, right panels in Fig. 3C) on the coalescent community. Strong and pervasive environmental selection against invaders, such as pH and anaerobic conditions [40], can inhibit entrants and their competition with residents (No. 8, Fig. 3C). This can lead to unsuccessful invasions and minimal perturbation of the resident community, weakening the competition effect over time. Conversely, resource addition in the soil can also undermine competition between invaders and residents when both require the same resource [38,86]. Furthermore, the confidence interval may rely on environmental heterogeneity, where lower heterogeneity reduces the importance of stochastic factors (No. 9, Fig. 3C). A crucial example is that increased soil homogeneity through multiple coalescence and vortex promotes homogenous selection, reducing stochasticity and decreasing bacterial alpha diversity [87]. However, enhanced environmental heterogeneity (e.g. soil aggregates and suspended particles in water [3,14]) may increase the stochasticity of interactions among species and between microbes and their environments by weakening the selection pressures present in resident communities. Moreover, niche release caused by other biotic and abiotic disturbances can reduce the competition between invaders and residents, providing more living space and available resources for invaders, at least in the short term (No. 10, Fig. 3C).

Higher-order interactions, which are mutually interfering interactions emerging in systems with more than two species, can impact the invader’s survival (No. 10, Fig. 3C). For instance, *Escherichia coli* can successfully invade cultures of either the alga *Chlamydomonas reinhardtii* (a phototroph) or the ciliate *Tetrahymena thermophila* (a predator) but fails to invade a community where both are present. This failure is due to the algal inhibition of bacterial aggregation, leaving the bacteria vulnerable to predation [88]. Additionally, inter-kingdom interactions with fungi, phages, soil macrobiota, protists, and plants can alter the competition between invaded bacteria and residents and influence invaders’ survival and coalescence impacts on resident communities (No. 10, Fig. 3C). For example, the inoculation of two protozoa species, *Rosculus terrestris* and *Cerocomonas lenta* can decrease the survival of bacterial inoculant (*Bacillus mycoides*) in soil via selective predation but increase the abundance of *Bacillus pumilus* by altering the resident community structure [89].

## Consequences of coalescence at the ecosystem level

The consequences of community coalescence at the ecosystem level should largely depend on its impacts at the community level. The potential modification of ecosystem functions and services relies on the direct impact of invaders or/and the indirect impact on resident communities [46]. A classic example is utilizing beneficial consortia in terrestrial ecosystems for better plant growth and productivity, where they can directly change the biogeochemistry of nutrient cycling [77,90] and indirectly alter the resident community composition and functions [91]. Like coalescence promoting the intended consequence of beneficial strains [77], however, microbial dispersal may facilitate the survival and pandemic of pathogenic strains across ecosystems [92]. At the same time, the spread and accumulation of antibiotic resistance might be reshaped, which is considered one of the greatest threats to human and animal health worldwide [93]. For example, river mainstreams in agricultural regions had a remarkably lower abundance of antibiotic resistance genes (ARGs) than branches after coalescence [94]. In terrestrial ecosystems, airborne ARGs from animal farms can disperse to a distance of 10 km along the wind direction and accumulate in the surrounding environments [95]. Therefore, it is important to reveal the resistance of local communities to pathogens and hosts of ARGs in the environment.

Community coalescence has implications for the functioning and stability (resistance and resilience) of entire coalescent communities and ecosystems [96]. Upon disturbances, microbial communities are often sensitive and cannot recover to their original status in terms of composition and functions [97]. Although disturbed resident communities can be partially restored by coalescing with the original community, these restorations have been shown to be incomplete and only valid for a short period in soil microcosms [54,83]. Therefore, microbial communities and their functioning might be continually reassembling at various speeds because of the unstoppable community coalescences that occur in natural ecosystems. This may partially elucidate why microbial biogeographic patterns appear weaker compared to those observed in plant and animal taxa [98]. As indicated by the single-species invasion in soil, the community assembly process might play an important role in community compositional stability and preventing invader colonization [99]. The reshaped resident community could start to exhibit altered biotic interactions, resource utilization patterns, and stability, which in turn may influence ecosystem-level processes such as nutrient cycling, energy flow, and primary productivity.

## Emerging questions and potential directions

Community coalescence plays an essential role in regulating soil microbial communities, which paves the way for understanding microbial community diversity and dynamics and points out an avenue for maintaining and managing ecosystem sustainability [35,100]. Next, we propose the questions that urgently need to be explored and possible research directions around coalescence mechanisms and consequences.

### Is competition between invaders and residents prevalent within and across ecosystems?

The interaction between invaders and resident residents stands as a pivotal factor shaping the fate of both groups. Prevailing research on invasion mechanisms suggests that antagonistic competition for available niches between invaders—whether individual species or communities—and native residents is commonly observed [31,38,86]. According to niche theory, it is reasonable to infer that available niches within natural, stable soil microbial communities are highly limited. Within resident communities, competition also exists widely, which can contribute to maintaining community stability [101] and the existence of competition-driven niche segregation among resident species [31] may contribute to mitigating excessive competition. Hence, does the level of competition between invaders and resident communities vary depending on the properties of the resident community itself? Could communities where exploitable niches be momentarily increased due to specific disturbances become more susceptible to invasions? Consequently, we propose experiments that coalesce the same source community with different resident communities to address these inquiries.

Considering the universality of microbial communities mixing among different ecosystems, it is also important to reveal whether there are differences in the mechanisms of community coalescence in different habitats. For example, compared to soil ecosystems, coalescence in aquatic systems, such as rivers and oceans, may occur more frequently and dynamically, potentially altering the primary mechanisms that regulate coalescence outcomes and their observation and prediction. Additionally, the nature of the communities coalescing, i.e. whether they are originally from the same ecosystem (i.e. either terrestrial or aquatic) or from different ecosystems (terrestrial coalescing with aquatic or vice versa), might also influence the relative importance of competition as a major mechanism driving the outcome of coalescence. For instance, communities from the same ecosystem may primarily coalesce due to competition for resources, given their similar life strategies and resource utilization. In contrast, the coalescence of communities from different ecosystems may involve weaker competition due to maladaptation, allowing other processes such as abiotic environmental selection, drift, and antibiotic release to play more dominant roles. However, little research has focused on these specific issues to date.

### To what extent are coalescence consequences predictable?

As discussed above, even if we propose a mechanistic model based on existing evidence (Fig. 3) and find that the outcome of community coalescence is predictable in the short term (such as 5 days and 60 days) [31], whether it can be predicted should depend on the underlying mechanisms controlling the coalescence and also the dynamic of coalescent communities. A crucial question is under which conditions the predictability of community coalescence is significantly reduced. Despite the theoretical possibility of invasional meltdown [62], it has rarely been reported due to microbial community coalescence in natural ecosystems. Subsequently, it is worth exploring which coalescence process is more likely to affect the predictability of the outcome. We speculate that the second step of the coalescence process may introduce stochasticity and greater heterogeneity in the environment itself or higher unevenness/frequency in the community mixing process may increase the unpredictability of consequences. We suggest that invaders’ survival and changes in resident communities need to be discussed separately within the context of coalescence. In doing so, we may shed further light on the importance of stochasticity in community assembly.

### How do we manipulate the survival rate of invaders?

Successfully controlling the establishment of invaders is important for the inoculation of beneficial microorganisms and the control of pathogenic microorganisms in agricultural ecosystems. Some studies have shown that artificially building synergistic relationships either between invaders and residents or among invaders can help the survival of inoculated microorganisms [63,77]. In addition to the biotic interactions, environmental conditions may also play an essential role. For example, a recent meta-analysis showed that inoculated consortia function better in soils rich in soil organic matter, available N and P, and with moderate pH [77]. Conversely, releasing niches in the recipient soil (e.g. via antibiotic treatment) can significantly increase invader survival [20]. These also indicate the pervasive role of competition in controlling coalescence consequences. Therefore, it is necessary to consider both the biotic and abiotic adaptations of invaders/inoculants during coalescence. One possible avenue is to link community metabolomics to coalescence feasibility, i.e. to select invaders relatively favored by metabolites produced by resident communities. Alternatively, exploiting the dormant ability of microorganisms might be a promising way to promote the survival and colonization of invaders. For example, the metabolically inactive strains can comprise the seed bank that acts as important buffers rescuing resiliency in ecosystems [102,103]. Moreover, there are cases where inoculated microorganisms can survive in the soil for long periods (longer than two decades) [104]. This raises two important questions. Firstly, what effect will the long-term presence of these invaders have on the ecosystem? Secondly, how can we manage and prevent any unneeded long-term impacts?

### How can we achieve community structural or functional restoration/manipulation through coalescence?

Community coalescence presents a theoretically promising approach for community restoration, either by preserving the existing community composition or by substituting original species with functionally equivalent species [2,102]. Indeed, introducing species in pre-disturbed soil can mitigate the impact of disturbances (such as heat shock, antibiotics, and oxidative stress) on soil community structure, thereby aiding in the restoration of community composition [54,83]. Similarly, by mixing diseased soil with biocontrol species [91] or healthy soil [105], the resident community can be restored/modified to suppress the *Fusarium* disease in plants. Similar ideas and efforts could be applied to key areas of marine ecosystems, such as to steer a healthier assemblage of coral microbiomes [106]. However, experimental efforts in these cases often involve direct mixing strategies, which may pose challenges in practical implementation within realistic production settings. Future research could explore the utilization and storage of “health” microbiomes for community coalescence or investigate the core taxa pivotal in the restoration of functional traits or redundancy, potentially considering their application for inoculation purposes.

### How do invaders and residents adapt and evolve during the coalescence process?

The phenomenon of community coalescence can potentially alter species’ evolutionary paths and gene flow within the community, albeit supported by limited evidence. In the case of invaders, their evolutionary prospects hinge upon their survival, which is intrinsically linked to the initial questions posed. Theoretically, invaders possess the potential to serve as sources of novel genes, transmitting them via horizontal gene transfer or viral activities [6]. Simultaneously, the selection pressure exerted by invaders can modify genetic exchange among original species, given their ability to suppress resident species within the community [31]. For instance, environmental stress induced by bleach (NaClO) did not significantly impact the overall plasmid transfer frequency but restricted the diversity of the resulting transconjugant pool [107], suggesting the importance of host diversity to microbial evolution. Consequently, the evolutionary trajectory of the community may undergo modifications through coalescence. However, the critical unknown remains whether such alterations will impact the community’s long-term functionality.

## Conclusion

Microbial community coalescence stands as an ever-present feature in natural ecosystems, now amplified to unprecedented levels by human activities. Its repercussions can simultaneously pose threats or offer remedies to community and ecosystem processes and functions. By contextualizing community coalescence within the community assembly framework, we dissect its key mechanisms, leveraging a model that integrates the biotic interactions between invaders and resident species. As we progressively uncover the complexities of these processes and understand the full range of consequences arising from community coalescence, we underscore the significance of this intricate phenomenon. Future research endeavors could elucidate the mechanisms underlying community coalescence and their ecological consequences across broad spatial and temporal scales within natural settings. Such insights will deepen our comprehension of community ecology and empower us to adeptly manage ecosystems facing ongoing environmental challenges.

## Data Availability

The datasets analyzed in the current study are available in the OSF repository via: https://osf.io/289hs/?view_only=e7f9829740d34cf6a8fffaa56632c674.
